# Serum uric acid and renal function in patients with type 1 diabetes: a nationwide study in Brazil

**DOI:** 10.1186/s13098-018-0324-7

**Published:** 2018-03-20

**Authors:** Marcela Haas Pizarro, Deborah Conte Santos, Bianca Senger Vasconcelos Barros, Laura Gomes Nunes de Melo, Marilia Brito Gomes

**Affiliations:** 1 Department of Internal Medicine, Diabetes Unit, State University of Rio de Janeiro (UERJ), Rio de Janeiro, Rio de Janeiro, Brazil; 2 Department of Ophthalmology, State University of Rio de Janeiro (UERJ), Rio de Janeiro, Rio de Janeiro, Brazil

**Keywords:** Type 1 diabetes, Serum uric acid, Glomerular filtration rate, Chronic renal disease, Albuminuria

## Abstract

**Background:**

Diabetes nephropathy is a microvascular complication associated with high morbidity and mortality in patients with type 1 diabetes, and its pathogenesis is not fully understood. Our aim was to evaluate the association between levels of serum uric acid and renal function assessed by glomerular filtration rate (GFR) and albuminuria in patients with type 1 diabetes.

**Methods:**

This is a multicenter, cross-sectional, observational study with 1686 patients, conducted between August 2011 and August 2014 in 14 public clinics from ten Brazilian cities. Renal function was estimated by CKD-EPI (adults) and by Schwartz (adolescents).

**Results:**

We analyzed 1686 patients, aged 30.1 ± 12.0, with 15.4 ± 9.3 years of duration of diabetes; 55.8% were female and 54.0% were Caucasians. Serum uric acid was related to renal function, with a mean of 4.8 ± 1.4 (in the normal renal function group) vs 5.2 ± 2.0 (GFR ≥ 60 ml/min and albuminuria) vs 6.5 ± 2.6 mg/dl (GFR < 60 ml/min). In the pooled group, multivariate analysis showed an inverse correlation between serum uric acid and GFR (r = − 0.316, p < 0.001) with a decrease of 4.11 ml/min in the GFR for every increase of 1 mg/dl in serum uric acid. Considering only patients with normal renal function (n = 1170), a decrease of 2.04 ml/min in the GFR for every increase of 1 mg/dl in Serum uric acid was noted using multivariate analysis.

**Conclusions:**

Patients with higher levels of serum uric acid have worse renal function, independently of HbA1c or duration of diabetes, which persisted even in patients with normal renal function. Further prospective studies are necessary to establish if patients with higher serum uric acid may have an elevated risk for developing chronic kidney disease.

**Electronic supplementary material:**

The online version of this article (10.1186/s13098-018-0324-7) contains supplementary material, which is available to authorized users.

## Background

Type 1 diabetes (T1D) is a chronic autoimmune disease which incidence is increasing worldwide [1]. Diabetes-related chronic complications resulting in elevated morbidity and mortality is still a concern in these patients [2]. Even though diabetic nephropathy (DN) is one of the most prevalent of such complications, its pathogenesis is not fully understood. Diabetes is responsible for 30–50% of all chronic kidney disease (CKD) [2] and is the main cause of renal failure requiring renal replacement therapy worldwide. This leads to a significant burden to public health [3]. Additionally, in T1D, the presence or severity of end-stage kidney disease was the main determinant of excess mortality, mainly from cardiovascular disease, independently of the use of statins or inhibitors of the renin-angiotensin system [4].

The physiopathology of chronic microvascular complications of T1D is complex, involving the interaction between genetic susceptibility, metabolic, and environmental factors. Many risk factors have already been associated with the development and progression of diabetic nephropathy, such as elevated HbA1c, duration of diabetes, presence of concomitant microvascular complications (especially retinopathy [5]) and elevated albumin excretion rate [6]. However, despite the increase in the use of renoprotective treatment and a more aggressive treatment of hypertension and diabetes, the risk of end-stage renal disease did not change over the years in a population of Caucasians patients with T1D that attended the Joslin Clinic [7].

Recent studies reveal that high serum uric acid (SUA) could also be a risk factor for DN [8, 9]. Studies disagree on whether uric acid has oxidant or antioxidant properties. In vitro studies show that uric acid may have antioxidant properties. SUA works as a scavenger of free radicals, reacting with a series of oxidants, especially peroxynitrite [10]. Other studies, both in vitro and in vivo, suggest that high levels of SUA may promote endothelial dysfunction [11], hypertension, and metabolic syndrome by inducing oxidative stress [10]. In fact, there is evidence that the use of drugs that lower serum uric acid can revert these conditions [12, 13].

Due to mechanisms not yet fully understood, patients with T1D usually have lower levels of SUA compared with healthy individuals or patients with T2D [14, 15]. One possible cause is glycosuria, leading to uricosuria mediated by activation of glucose transporter 9 (GLUT 9) isoform 2 on the apical membrane of the proximal tubule [14]. However, high-normal levels of SUA in patients with T1D have been recently associated to higher prevalence of DN [8, 9]. Studies found an 80% elevation in the risk of developing albuminuria per 1 mg/dl increase in SUA concentration, even in high-normal ranges of SUA [16]. Higher levels of SUA were also associated with a reduced glomerular filtration rate (GFR) estimated by serum Cystatin C [17]. Another study showed that the use of allopurinol was associated with a reduction of SUA, systolic and diastolic blood pressure, and urinary albumin excretion rate in patients with T2D [18], supporting the association between elevated SUA and DN. Studies with the use of allopurinol are still being conducted in patients with T1D [19]. Since SUA is a potentially modifiable risk factor for DN, a complication associated with high morbimortality, further investigation is needed.

The goal of this study is to examine the association between levels of SUA and the presence of CKD, determined by the GFR and albuminuria, in patients with T1D in a multicenter, cross-sectional, observational study.

## Methods

This is a multicenter, cross-sectional, observational study performed by the Brazilian type 1 Diabetes Study Group (BrazDiab1SG) with 1760 patients with T1D, from 14 public clinics of the secondary and tertiary care levels located in ten cities among all geographical areas of Brazil. Data were collected between August 2011 and August 2014. The methods have been described previously [20].

Briefly, all patients received health care from the National Brazilian Health Care System. Each clinic provided data from at least 50 patients with T1D that attended the center. An endocrinologist followed all patients in secondary or tertiary centers. Inclusion criteria were: patients with 13 years of age or older, medical follow-up for at least 6 months at the respective center, patients with at least 5 years of diagnosis of T1D, and diagnosis of T1D by a physician. T1D was diagnosed based on the presence of classic clinical presentation at the moment of the diagnosis, such as polyuria, weight loss, polydipsia, and the need for continuous insulin use since the moment of the diagnosis. Exclusion criteria were: pregnancy or lactation at the moment of inclusion, history of renal transplant, and acute infection or ketoacidosis in the 3 months before the recruitment.

Patients between 13 and 19 years of age were classified as adolescents, and patients older than 19 years were considered adults, based on the American Diabetes Association (ADA) criteria [21].

The study was approved by the ethics committee of Pedro Ernesto University Hospital (State University of Rio de Janeiro) and by the local ethics committee of each center. All participants or their parents signed the informed consent form.

Patients with disease duration greater than or equal to 5 years were submitted to a screening of chronic complications related to T1D, such as screening for microvascular disease (retinopathy, nephropathy, and peripheral neuropathy).

The following variables were obtained using a questionnaire during a clinical visit: gender, current age (years), race, age at diagnosis of T1D, duration of diabetes (years), smoking status, consumption of alcohol, daily dose of insulin, use of other medications, and associated diseases.

The following clinical variables were evaluated: weight (kg), height (m), body mass index [BMI (kg/m^2^)], systemic blood pressure, heart frequency, and abdominal circumference.

Fasting plasma glucose, levels of HbA1c, creatinine, urea, triglycerides, total cholesterol, high-density lipoprotein (HDL) cholesterol, low-density lipoprotein (LDL) cholesterol levels measured during the last clinical visit were obtained from the patient’s medical record. The levels of HbA1c and SUA were measured in a single center. HbA1c was measured using high-performance liquid chromatography (HPLC, Bio-Rad Laboratories, Hercules, California, USA). Fasting plasmatic glucose, HDL cholesterol, total cholesterol, triglycerides, and SUA were measured using enzymatic techniques. SUA was measured using an uricase-based commercial kit (BioSystem) with results expressed in milligrams per deciliter (mg/dl) and normal range between 3.5–7.2 mg/dl in men and 2.6–6.0 mg/dl in women. Friedewald’s equation was used to calculate LDL cholesterol values [22]. Creatinine was measured using a colorimetric assay kit (Biosystems).

Albuminuria was measured from a morning urine sample. This procedure was repeated twice with a minimal interval of 1 week and maximal of 6 months between each collected sample. The dosage of urinary albumin was evaluated by immunoturbidimetry and the results were expressed as mean (mg/dl). Albuminuria was defined as albuminuria ≥ 30 mg/dl [2]. Samples with hematuria or urinary infection were excluded based on a urinalysis performed before the collection of urine for albuminuria. All patients were instructed to avoid physical exercise before the collection of the urine sample.

Renal function was estimated by the CKD-EPI equation [23] in adults and by the Schwartz formula in adolescents [24] and expressed as glomerular filtration rate (GFR) in milliliters per minute per 1.73 m^2^ (ml/min). Patients were divided according to levels of renal function into three groups for comparison purposes. Group 1 included patients with a GFR ≥ 60 ml/min with absence of albuminuria. Group 2 included patients with a GFR ≥ 60 ml/min and the presence of albuminuria. Finally, patients in group 3 had a GFR < 60 ml/min with or without albuminuria.

### Statistical analysis

Continuous variables are presented as the means ± standard deviations (SD), and medians and interquartile range (IQR) that were also used to perform a boxplot graph. Frequencies and percentages were used to present categorical variables. Differences between categorical variables were assessed using Chi square and differences between independent continuous variables were assessed using Student’s t tests or ANOVA with Sidak correction. A Pearson’s correlation coefficient was calculated when applicable. A partial correlation was run to determine the relationship between SUA and GFR while controlling for mean albuminuria in the pooled group. All variables included in the multivariate analysis had a significant Pearson coefficient, except for economic status, smoking, and HDL cholesterol (data not shown). We performed a multivariate linear regression using Generalized Linear Models (GLM) to explore the association between SUA and renal function expressed in GFR as the dependent variable. Adjustments were made for potential confounders, such as: HbA1c, BMI, gender, duration of diabetes, ethnicity, years of education, economic status, smoking, systolic and diastolic blood pressure, use of diuretics, statin or inhibitors of the renin-angiotensin system, LDL and HDL cholesterol, and mean albuminuria. The result was not adjusted for age because age is already included in the CKD-EPI equation. The tests were performed initially in all patients (pooled group) and then in a subgroup with normal renal function. All statistical analyses were performed with Statistical Package for Social Sciences (SPSS) 24.0 for Mac. Confidence intervals (95%) were expressed when indicated. A two-sided *p* value less than 0.05 was considered significant.

## Results

### Participants’ demographic and clinical characteristics according to levels of renal function

Among 1760 patients, we excluded 62 patients because data were not available, and twelve because they had a history of renal transplant. The study, therefore, included 1686 (96%) patients. Clinical characteristics of the patients included in the study are summarized in Table 1. Clinical and demographic data of the studied population stratified into levels of renal function are summarized in Table 2.

**Table 1:** Clinical and demographic data of patients in the pooled group

Variables	All patients
N	1686
Gender, n (% female)	942 (55.8)
Age, year	30.1 ± 12.0
Duration of diabetes, year	15.4 ± 9.3
Years of study	12.2 ± 3.8
Ethnicity, n (%)	
Caucasian	911 (54)
Non-caucasian	774 (46)
Economic status, n (%)	
High	52 (3.1)
Medium	764 (45.3)
Low	815 (48.3)
Very low	55 (3.3)
Geographic region, n (%)	
Southeast	787 (46.7)
South	223 (13.2)
North/Northeast	470 (27.9)
Mid-West	206 (12.2)
Diabetes management and glycemic control	
Insulin dose (units/kg/day)	0.8 ± 0.4
HbA1c (%)	9.0 ± 2.1
HbA1c (mmol/mol)	75 ± 23
Cardiovascular risk factors	
Cigarette smoking, n (%)	89 (5.3)
Hypertension, n (%)	294 (17.4)
Dyslipidemia, n (%)	359 (21.3)
Systolic blood pressure (mmHg)	121.6 ± 16.2
Diastolic blood pressure (mmHg)	75.0 ± 10.4
Body mass index (kg/m^2^)	24.1 ± 4.1
Serum uric acid (mg/dl)	5.1 ± 1.9
Mean albuminuria (mg/dl)	59.3 ± 309.4
Waist circumference (cm)	82.7 (11.5)

*y* years, data are presented as number (percentage) or mean ± SD

**Table 2:** Characteristics of patients with type 1 diabetes according to groups of renal function

Variables	Groups			p value
	Normal renal function	GFR ≥60 ml/min and albuminuria	GFR < 60 ml/min	
n = 1686	1170	246	270	
Age, (year)	28.7 ± 11.7	28.1 ± 10.0	37.9 ± 11.7*	p < 0.001
Gender, (% female)	631 (53.9)	125 (50.8)	186 (68.8)*	p < 0.001
Duration of diabetes, (year)	14.1 ± 9.1	15.2 ± 7.5	20.8 ± 9.8*	p < 0.001
Years of study, (year)	12.3 ± 3.9	11.9 ± 3.5	11.8 ± 3.8	p = 0.086
Insulin dose, (units/kg/day)	0.86 ± 0.36	0.94 ± 0.46*	0.79 ± 0.36*	p < 0.001
Cigarette smoking, (%)	58 (5.0)	15 (6.1)	16 (5.9)	p = 0.233
Hypertension, (%)	130 (11.1)	45 (18.3)*	119 (44)*	p < 0.001
Dyslipidemia, (%)	203 (17.4)	51 (20.8)	105 (39)*	p < 0.001
Systolic blood pressure (mmhg)	119.1 ± 14.1	124.2 ± 16.1*	129.5 ± 20.7*	p < 0.001
Diastolic blood pressure (mmhg)	73.3 ± 9.4	78.1 ± 10.6*	79.6 ± 12.3*	p < 0.001
BMI (kg/m^2^)	24.0 ± 4.0	24.1 ± 4.0	24.9 ± 4.3	p = 0.005
HbA1c (%)	8.9 ± 2.0	9.9 ± 2.5*	8.9 ± 2.0	p < 0.001
HbA1c (mmol/mol)	73.2 ± 22.2	84.8 ± 27.2*	73.5 ± 21.3	p < 0.001
Serum uric acid (mg/dl)	4.8 ± 1.4	5.2 ± 2.0*	6.5 ± 2.6*	p < 0.001

*GFR* Glomerular filtration rate (ml/min), *SD* standard deviation, *y* years, data are presented as number (percentage), mean ± SD. ANOVA was used for continuous variables and Chi square test for categorical variables

* p < 0.05 vs Normal Renal function group

The duration of diabetes was longer and the diagnoses of hypertension and dyslipidemia were more prevalent in patients with albuminuria or lower renal function (p < 0.001). The distribution of gender varied according to different levels of renal function, with the percentage of female patients increasing from 53.9 to 69% when comparing patients with normal renal function and GFR < 60 ml/min, respectively (p < 0.001).

### Serum uric acid and renal function in the pooled group

**Fig. 1:**
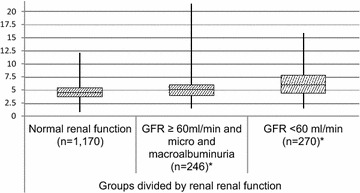
Boxplot of serum uric acid levels in groups stratified by renal function

Higher SUA was observed in patients with GFR < 60 ml/min in comparison to patients in other levels of renal function. A box plot graph (Fig. 1) illustrates the medians of SUA levels in groups, stratified by renal function. Median SUA was 4.8 ± 1.4 mg/dl in the group with normal renal function, 5.2 ± 2.0 mg/dl in patients with a GFR ≥ 60 ml/min and micro or macroalbuminuria, and 6.5 ± 2.6 mg/dl in patients with GFR < 60 ml/min (p < 0.001). A negative correlation was observed between the levels of SUA and GFR (r = − 0.316, p < 0.001). There was a negative partial correlation between SUA and GFR while controlling for mean albuminuria (− 0.302, p < 0.001).

### Multivariate analysis in the pooled group

A multivariate regression analysis revealed that levels of SUA were negatively correlated with the GFR, even after adjustment for the confounding factors (p < 0.001). Every 1 mg/dl increase in SUA was associated with a decrease of 4.11 ml/min in the GFR. The final adjusted model using Generalized Linear Models is described in Additional file 1: Table S1.

### Serum uric acid in patients with normal renal function

These 1170 patients had very similar clinical and demographic characteristics when compared to the pooled population. No difference was noted in the HbA1c, with 9.0 ± 2.1% (75 ± 23 mmol/mol) in the overall studied population and 8.9 ± 2.0% (73.2 ± 22.2 mmol/mol) in the group with normal renal function. Patients with normal renal function had a lower prevalence of hypertension (11.1% vs 17.4%) and dyslipidemia (17.4% vs 21.3%), and a lower SUA (4.8 ± 1.4 vs 5.1 ± 1.9 mg/dl) when compared to patients in the pooled group. SUA had a negative correlation with the levels of renal function (r = − 0.093, p: 0.001) in these patients with normal renal function.

### Multivariate analysis in the group of patients with normal renal function

In patients with normal renal function, every 1 mg/dl increase in SUA was associated with a decrease in 2.04 ml/min in the GFR. This association persisted even when corrected by the confounding factors. The final adjusted model using Generalized Linear Models is described in Additional file 1: Table S2.

## Discussion

Our cross-sectional study indicates that patients, from an admixed and multi-ethnic population, with worse renal function have higher levels of SUA, independent of HbA1c, duration of diabetes and other demographic and clinical variables. Patients with normal GFR (GFR ≥ 60 ml/min) and the presence of albuminuria have a higher SUA when compared to those with absence of albuminuria. The partial correlation used to determine the relationship between SUA and GFR while controlling for mean albuminuria showed that albuminuria had very little influence in the relationship between SUA and GFR. This association of SUA and GFR persists even in patients with normal renal function without albuminuria, suggesting that patients with an elevated SUA may have a higher chance of developing CKD.

The causality relationship between SUA and DN is still controversial. Some studies have already confirmed the association between high-normal SUA and the development or progression of DN. A study conducted with adolescents with T1D showed that a higher SUA was correlated with a lower GFR, even when corrected for gender, HbA1c, duration of diabetes, and other confounding factors [25]. This is relevant because adolescents had lower levels of SUA when compared to healthy control subjects [25] and generally don’t have other comorbidities such as hypertension or dyslipidemia that are also related to the development of diabetic nephropathy. In our study, the majority of patients were adults. As expected, we found a higher prevalence of comorbidities such as hypertension and dyslipidemia. However, similar results concerning the relationship between SUA and GFR were observed despite correction for these and other confounding factors.

A study conducted with 355 patients with T1D, with 6 years of follow-up, revealed that for each 1 mg/dl increase in serum uric acid, there was a 40% increase in the risk of developing early GFR loss. They defined progressive renal decline as a loss higher than 3.5 ml/min/year of estimated GFR measured by cystatin. There was a linear increase in this risk of early GFR loss across the normal range of SUA levels [9]. Our study showed similar results, even considering that it is a cross-sectional study. There was a decrease of 4.11 ml/min with every increase in 1 mg/dl in SUA, and even patients with normal renal function had a decrease in the GFR with an elevation of SUA. However a recent FINISH cohort study with a follow-up of 3895 patients during 7 years have suggested that serum uric acid was not causally related to diabetic nephropathy but instead, appears to be a downstream marker of kidney damage [26].

Studies show conflicting results as to whether uric acid is a biomarker of renal dysfunction or if it has an etiologic role in the progression of CKD. For instance, patients with CKD that were randomized to receive treatment with allopurinol 100 mg/day had a slower progression of renal disease in comparison with the control group, independently of age, gender, and albuminuria. They also had lower risk of cardiovascular events [27]. Another study showed that hyperuricemic rats had a worse renal function, a higher prevalence of proteinuria, hypertension, and thickening of preglomerular vessels, mediated by the activation of the renin angiotensin system by uric acid. This system could be responsible for an increased glomerular and systemic pressure and also for a direct fibrogenic effect on renal and vascular cells [28]. These effects can be attenuated with the use of their inhibitors [29]. However, in our study, SUA was correlated with a worse GFR, despite the use of inhibitors of the renin angiotensin system, suggesting that other mechanisms could be involved in the renal dysfunction associated with a high SUA. Another possible mechanism could be an impaired nitric oxide production, associated with endothelial dysfunction. Previous data from our group showed similar results, with SUA, even in the upper limit of normality, functioning as a strong predictor of impaired microvascular endothelial function in patients with T1D [30].

A particular strength of our study is the population-based ascertainment of diabetes cases in a large sample of Brazilian patients with T1D, from a wide range of ethnic groups, from all geographic regions of the country. All participating centers followed a uniform and standardized protocol. Similar to other population-based studies, we used a clinical definition of T1D assigned by healthcare providers that was applicable to all patients.

We found that patients with a normal GFR but with the presence albuminuria had a higher SUA when compared to those with normal renal function. However, the difference between the two groups was modest and should be interpreted with caution.

Our study has some limitations. First, it was a cross-sectional study so we cannot establish causality between levels of SUA and CKD. We cannot determine if elevated SUA is only a biomarker of the decline of renal function or is also a risk factor for CKD. SUA is eliminated mainly by the kidneys, so it increases as a result of the decline in the GFR. However, the rise in SUA in CKD is generally mild because of enhanced uric acid enteric excretion and a decrease in its production, by reduced xanthine oxidase activity [31]. In the subgroup of patients with normal renal function, without albuminuria, we still found an independent relationship between higher levels of SUA and GFR. This suggests that the elevation of SUA cannot be entirely attributed to the decrease of uric acid filtration rate that accompanies the worsening of renal function. Second, we only included patients that were followed in secondary and tertiary centers, so patients treated in primary clinics were excluded. This probably had little impact in our study since the majority of patients with T1D are followed in secondary and tertiary centers in Brazil. Third, we used the measurement of albumin concentration from a morning sample of urine instead of measurement in urine collected in 24 h. However, studies show that the urinary albumin concentration in a random spot urine is a reliable predictor of diabetic nephropathy and cardiovascular events [32]. Finally, we evaluated albuminuria with a spot urine sample measuring albumin alone, without simultaneously measuring urine creatinine, consequently elevating the risk of false-negative and false-positive results. We decided to measure only the albumin concentration because it is less expensive considering the large sample group analyzed in this study and because it has already been described as a reliable measure in previous studies [32].

## Conclusions

To our knowledge, this is the first study to establish the association between levels of SUA and renal function in patients with T1D in Brazil. We found an association between a decrease in GFR and higher levels of SUA, even when patients had a normal renal function, independently of other confounding factors such as HbA1c, gender, and duration of diabetes. Further prospective studies are necessary to establish if patients with a higher SUA may have an elevated risk for developing CKD. Ongoing randomized, controlled trials with drugs that lower SUA in T1D will establish the relationship between SUA and renal function.

## Additional file

**Additional file 1: Table S1.** Adjusted regression model for GFR in the pooled group (n=1686). **Table S2.** Adjusted regression model for GFR in patients with normal renal function (n=1170).
