# Supplementary material for: Serum uric acid and renal function in patients with type 1 diabetes: a nationwide study in Brazil
Source: Diabetol Metab Syndr. 2018 Mar 20;10:22. doi: 10.1186/s13098-018-0324-7 (PMC5859721; doi:10.1186/s13098-018-0324-7)
Supplement: Supplementary file 1 — Additional file 1: Table S1. Adjusted regression model for GFR in the pooled group (n=1686). Table S2. Adjusted regression model for GFR in patients with normal renal function (n=1170). [file 13098_2018_324_MOESM1_ESM.docx]

| Table S1: Adjusted regression model for GFR in the pooled group (n=1,686) | | | | | |
| --- | --- | --- | --- | --- | --- |
| Parameter | B | Std. Error | 95% Confidence interval for B | |  |
|  |  |  | Lower | Upper | p-value |
| (Intercept) | 161.3 | 8.76 | 144.2 | 178.5 | 0.000 |
| Gender, female | -14.17 | 1.43 | -16.97 | -11.37 | 0.000 |
| Serum uric acid (mg/dl) | -4.11 | 0.39 | -4.87 | -3.35 | 0.000 |
| Use of statins | -2.25 | 1.75 | -5.68 | 1.18 | 0.198 |
| Use of inhibitors of the renin-angiotensin system | -2.55 | 1.71 | -5.90 | 0.79 | 0.134 |
| Use of diuretics | -13.38 | 2.62 | -18.51 | -8.25 | 0.000 |
| Non caucasian | 1.14 | 1.34 | -1.48 | 3.75 | 0.394 |
| High economic status | 10.17 | 5.51 | -0.62 | 20.96 | 0.065 |
| Medium economic status | 4.43 | 4.00 | -3.41 | 12.27 | 0.268 |
| Low economic status | 2.92 | 3.90 | -4.72 | 10.57 | 0.453 |
| Very low economic status | 0^a^ |  |  |  |  |
| Non- smokers or ex-smokers | 0.40 | 2.88 | -5.25 | 6.05 | 0.889 |
| Educational years, y | -0.84 | 0.189 | -1.21 | -0.47 | 0.000 |
| BMI | -0.17 | 0.17 | -0.50 | 0.16 | 0.322 |
| Mean systolic blood pressure (mmHg) | -0.21 | 0.064 | -0.34 | -0.09 | 0.001 |
| Mean diastolic blood pressure (mmHg) | -0.05 | 0.09 | -0.22 | 0.13 | 0.599 |
| Duration of diabetes, y | -0.69 | 0.08 | -0.85 | -0.53 | 0.000 |
| HbA1c (%) | 1.21 | 0.33 | 0.58 | 1.85 | 0.000 |
| LDL cholesterol | -0.036 | 0.016 | -0.07 | -0.004 | 0.027 |
| HDL cholesterol | 0.012 | 0.035 | -0.056 | 0.081 | 0.723 |
| Albuminuria (mg/dl) | -0.006 | 0.0021 | -0.010 | -0.002 | 0.599 |
| (Scale) | 582.405b | 21.8419 | 541.132 | 626.827 |  |

0a: reference ,SD: standard deviation, GFR: Glomerular filtration rate, BMI: body mass index, LDL: low-density lipoprotein, HDL: high-density lipoprotein

| Table S2: Adjusted regression model for GFR in patients with normal renal function (n=1,170) | | | | | |
| --- | --- | --- | --- | --- | --- |
|  | B | Std. Error | 95% Confidence interval for B | |  |
| Parameter |  |  | Lower | Upper | p-value |
| (Intercept) | 143.4 | 10.1 | 123.6 | 163.2 | 0.000 |
| Gender, female | -9.77 | 1.54 | -12.78 | -6.76 | 0.000 |
| Serum uric acid (mg/dl) | -2.04 | 0.52 | -3.06 | -1.01 | 0.000 |
| Use of statins | -1.62 | 1.98 | -5.51 | 2.27 | 0.414 |
| Use of inhibitors of the renin-angiotensin system | -3.27 | 2.07 | -7.33 | 0.78 | 0.113 |
| Use of diuretics | -5.37 | 3.46 | -12.15 | 1.4 | 0.120 |
| Non caucasian | -0.15 | 1.39 | -2.87 | 2.57 | 0.914 |
| High economic status | 3.81 | 5.71 | -7.40 | 15.01 | 0.505 |
| Medium economic status | 3.12 | 4.39 | -5.49 | 11.72 | 0.478 |
| Low economic status | 1.38 | 4.289 | -7.03 | 9.79 | 0.747 |
| Very low economic status | 0^a^ |  |  |  |  |
| Non- smokers or ex-smokers | -4.30 | 3.26 | -10.70 | 2.10 | 0.188 |
| Educational years, y | -0.81 | 0.198 | -1.200 | -0.422 | 0.000 |
| BMI | -0.50 | 0.187 | -0.865 | -0.132 | 0.008 |
| Mean systolic blood pressure (mmHg) | -0.141 | 0.068 | -0.275 | -0.007 | 0.039 |
| Mean diastolic blood pressure (mmHg) | 0.058 | 0.094 | -0.127 | 0.243 | 0.537 |
| Duration of diabetes, y | -0.523 | 0.085 | -0.69 | -0.36 | 0.000 |
| HbA1c (%) | 1.54 | 0.351 | 0.85 | 2.23 | 0.000 |
| LDL cholesterol | -0.025 | 0.019 | -0.06 | 0.011 | 0.178 |
| HDL cholesterol | 0.012 | 0.038 | -0.063 | 0.086 | 0.762 |
| (Scale) | 487.149b | 20.8576 | 447.937 | 529.793 |  |

0a: reference, SD: standard deviation BMI: body mass index, LDL: low-density lipoprotein, HDL: high-density lipoprotein
